# Sex-Dependent Outcome of Hepatitis B and C Viruses Infections: Synergy of Sex Hormones and Immune Responses?

**DOI:** 10.3389/fimmu.2018.02302

**Published:** 2018-10-08

**Authors:** Anna Ruggieri, Maria Cristina Gagliardi, Simona Anticoli

**Affiliations:** Center for Gender Specific Medicine, Istituto Superiore di Sanità, Rome, Italy

**Keywords:** sex, HBV, HCV, estrogens, androgens, progesterone, innate immune response, adaptive immune response

## Abstract

Hepatitis B virus (HBV) and hepatitis C virus (HCV) are hepatotropic viruses that differ in their genomic content, life cycle and molecular prognosis. HBV and HCV establish chronic lifespan infections that can evolve to fibrosis, cirrhosis and hepatocellular carcinoma (HCC). This malignant liver cancer affects more commonly male patients than females, with a male-to-female incidence ratio of <Capword>2</Capword>:1 up to 7:1. Sex significantly contributes to shape the immune responses, contributing to differences in the pathogenesis of infectious diseases, in males and females patients. Females usually develop more intense innate, humoral and cellular immune responses to viral infections and to vaccination compared to male subjects. Sex hormones, in turn, differentially affect the immune responses to viruses, by specific binding to the hormone receptors expressed on the immune cells. In general, estrogens have immune-stimulating effect, while androgens are immune-suppressing. However, sex hormones, such as androgen, can also directly interact with HBV genome integrated into the cell nucleus and activate transcription of HBV oncoproteins. On the other side, estradiol and estrogen receptors protect liver cells from inflammatory damage, apoptosis and oxidative stress, which contribute to fibrosis and malignant transformation preceding HCC. In HCV-associated cirrhosis and HCC the decreased expression of estrogen receptor alfa (ERα) in male patients may explain the worse outcome of HCV infection in men than in women. The synergistic action of male and female sex hormones and of immune responses, together with viral factors contribute to the mechanism of sex/gender disparity in the outcome and progression of hepatitis viruses infection.

## Introduction

Men and women are extremely different in health and disease. Women usually live longer than men but not healthier; in fact, women are often sicker than men. In addition, sex-related differences in the frequency of side effects have been reported for several drugs, with women experiencing more adverse events than men (1). The higher rate of side effects in women than in men may be due, at least in part, to the fact that results from clinical trials derive mainly from male subjects, being women inadequately represented in clinical trials (2). In the last 18 years gender-specific medicine has been recognized as the study of how diseases differ between sexes in terms of susceptibility, prevention, clinical manifestations, therapy, prognosis and mechanisms of pathogenesis (3). In this context, viral infections have been recognized to differ between males and females for prevalence, intensity, outcome and pathogenetic mechanisms (4). In this minireview, we introduce some mechanisms that determine sexual dimorphism in immune function in males and females. We then discuss the impact of the synergy between sex hormones and sexually dimorphic immune responses on pathogenesis during Hepatitis B (HBV) and Hepatitis C (HCV) viral infections.

## Sex disparity in immune responses to viral infections

It is well-known that sexual dimorphism occurs in humans and animals with regard to immune responses and viral infections (4). Female individuals usually are less susceptible to viral infections than males, since they mount a more efficient, intense and prolonged immune response, either innate, as well as humoral and cell-mediated (5, 6). The innate immune response is the first line of defense against viruses and it is mediated by Toll-like receptors (TLRs), retinoic acid-inducible gene I-like receptors (RIG-I) and nucleotide oligomerization domain-like receptors (NOD-like receptors). These, named pattern recognition receptors (PRRs), recognize viral components (such as DNA, dsRNA, ssRNA, and viral proteins) and activate production of type 1 interferon (IFN) and inflammatory cytokines (IL-1, TNFs). In rodents and in humans expression of TLRs (such as TLR7) as well as number of monocytes, macrophages and dendritic cells, that are innate immune response players, have been reported to be significantly higher in females than in males (7, 8), thus accomplishing the more intense inflammatory responses in female subjects than in males (9).

In general, once a viral infection is established, the activation, by the Antigen Presenting Cells (APCs), of adaptive immune responses and of B cells, with subsequent rise of the antibodies specific for viral antigens, in most cases is greater in female animals and humans compared to males (10). In addition, females have higher number of CD4^+^T cells than males, that induces a greater number of T cells activated by viral antigens engagement of the T cell receptor (9–13); moreover, stronger cytotoxic T cell activity along with overexpression of antiviral and pro-inflammatory genes, many of which have estrogen response elements in their promoters, have been reported in women (14).

In most viral infections, following viral clearance, when the immune system returns to the homeostasis, basal immune responses are higher in females than in males. This can result in a higher risk of developing immunopathologies associated to viral infections in female individuals. In contrast, the lower antiviral immune responses at homeostasis in males can be responsible for the increased risk to undergo to persistent viral infections (9).

Based on this, it is deductible that female are less prone than males to be virus infected, due to their more effective antiviral immune defenses, but more frequently they develop more severe symptoms, due to the more intense inflammatory responses (9).

The immunological dimorphism between sexes, besides being shaped by the individual genetic background (15, 16), is mostly regulated by sex steroids hormones, particularly by estrogens, progesterone and androgens, that affect function of the immune cells. Due to the expression of the sex hormone receptors on immune cells, including lymphocytes, monocytes and dendritic cells, the interaction of sex hormones and immune cells affects release of cytokines and chemokines, which determine differentiation, maturation and proliferation of the immune cells (17).

Presence of different sex hormones, which circulate at different levels in males and females, makes sense of the fact that the immune responses are differentially modulated in individuals of different sexes (18). There are three classes of sex hormones: androgens (testosterone), estrogens (17-beta-estradiol) and progesterone. The level of estrogens in females fluctuates during menstrual cycle and declines with menopause; in males the testosterone level is stable up to almost 60 years of age, before age declining. Consequently, sex disparity in immune responses to viral infections may vary with aging.

Several studies have been published so far to clarify the various roles of estrogens on immune system, whereas much less is known about the roles of androgens (19–21). In general, testosterone has been demonstrated to have suppressive effect on the immune function, either in animal models and in human trials (21); conversely the effect of estrogen varies depending on their levels and on the immune measure used (20).

Androgens have been shown to suppress pro-inflammatory responses in rodents, by increasing production of anti-inflammatory cytokines (IL-10, TGF-β) (22–24). In humans, androgens deficiency in men has been reported to induce increased levels of inflammatory cytokines (IL-1β, IL-2, and TNF), an increased CD4^+^/CD8^+^ T cell ratio and higher antibodies titers, compared to male subjects with normal level of testosterone (25, 26). Actually, several studies (21, 27) have indicated that androgens exert an overall inhibitory effect on differentiation of Th1 arm of the immune system, with consequent reduced production of IFN-γ that may explain the enhanced susceptibility to viral infections in males than in females (28).

Estrogens (17β-estradiol) act by binding to the estrogen receptors (ER) -alfa or -beta, which are expressed differentially among the subsets of immune cells: ERα is highly expressed on T cells and ERβ highly expressed on B cells (19). Estrogens affect different activity of the innate and adaptive immune responses, and they have opposite effects on the immune system based on their concentration. In humans, low doses of estrogens have been reported to induce monocytes differentiation into inflammatory DCs with consequent high production of IL-4 and IFN-α. Conversely, high doses of estrogen have inhibitory activity on innate and pro-inflammatory immune responses (19, 29).

In addition, the low level of estrogens activates Th1-type and cell-mediated immune responses, whereas high levels enhance Th2-type responses and humoral immune responses in diverse species and *in vitro*. Treg cell populations are also positively affected by estrogens either in mouse model and in women (30). At physiological concentration estrogens also stimulate humoral response to viral infections, by inducing higher levels of antibodies and activating antibodies-producing cells. From the above it is deductable that the fluctuation of the estrogen levels during the menstrual cycle in female subjects can make women differently immune-reactive before ovulation, when the antibody levels are highest (31).

Progesterone's effect on immune system is similar to androgen's immune suppression of both innate and cell mediated immune responses. It is known that progesterone suppresses Th1 response and favors the Th2 cytokines production, inhibits cytotoxic T cells and modulate function of NK cells (5).

## Sex disparity in hepatitis B virus (HBV) and hepatitis C virus (HCV) infections

HBV and HCV are two hepatotropic viruses belonging, respectively, to Hepadnaviridae (HBV) and Flaviviridae (HCV) families, that differ in their genomic content, life cycle and molecular prognosis. HBV is the DNA virus that has the ability to integrate into host cell DNA, establishing persistent infection and whose replication cycle involves an RNA intermediate. HCV is the RNA virus that replicates in host cell cytoplasmic membranous webs (vescicle-like cytoplasmic membranes). Escaping from innate and adaptive immune responses is the main mechanism involved in establishment of persistent HCV infection (32).

Both viruses are responsible for chronic infections and represent a major risk factor for the development of hepatocellular carcinoma (HCC). Male sex is a risk factor for HBV and HCV prevalence and for HCC development subsequent to HBV and/or HCV infection. HBV-associated HCC develops more frequently in men than in women, with a female/male ratio ranging from 1:4 to 1:7 (33). In addition, female HBV carriers have lower viral loads than male carriers (34, 35) and the prevalence of serum HBV surface antigen (HBsAg) has been reported higher in men than in women (36). Some studies suggested that high levels of serum testosterone could associate with an increased risk of HCC development in male carriers of HBV (37). This and similar observations suggested that HBV infection and pathogenesis could be regulated by sex hormones. As reported above, male and female sex hormones affect the release of inflammatory cytokines in opposite way, as estrogens induce pro-inflammatory cytokine whereas androgens suppress pro-inflammatory responses, and if this occurs in the HCC microenvironment it can contribute to the epigenetic changes responsible for malignant transformation in different or opposite ways between sexes.

As described in the previous paragraph, the innate immunity response is the first line of defense against viral infections. The impact of sex differences and, in particular, of sex hormones on innate immune response to HBV are largely unknown, due, at least in part, to the lack of adequate models. A little more is known regarding gender-related differences in adaptive immune response to HBV infection. After prophylactic vaccination against HBV, women have higher anti-HBs antibody titer than men (38). In addition, a more frequent seroconversion to HBeAg (HBV e antigen) and HBsAg antibodies has been reported in female HBV chronic carriers than in males (39). According to a study conducted in mouse models of acute and persistent HBV infection, sex-related discrepancies in the adaptive response to HBV infection may be explained by different CD8^+^-T cells activity. In both murine models higher CD8^+^-T cells activity has been reported in females than in males and correlated to lower number of intrahepatic Treg cells in female mice than in male ones (40). However, the role of androgens and estrogens in regulating T-cells response to HBV infection is still unclear, and only some clues are available. For example, susceptibility to chronic HBV infection has been associated to a particular ERα polymorphism; one possible explanation is that this polymorphism affects ESR1 (Estrogen Receptor 1) gene transcription with the consequence of a defective response of immune cells to estrogens (41). Given the reported effect of sex hormones on immune system, it is reasonable to speculate that this can also account for the sex different susceptibility to HBV infection. Antiviral immune response modulation by sex hormones may also contribute to explain HCC prevalence in male gender, as in the case of chemically induced HCC that is worse in male than in female mice, due to the increased production of IL-6 by Kupffer cells in the males liver (42). Same Authors showed that estrogens transcriptionally inhibited IL-6, through reduction of Myd 88-dependent induction of NF-κB (42, 43).

**Figure 1:**
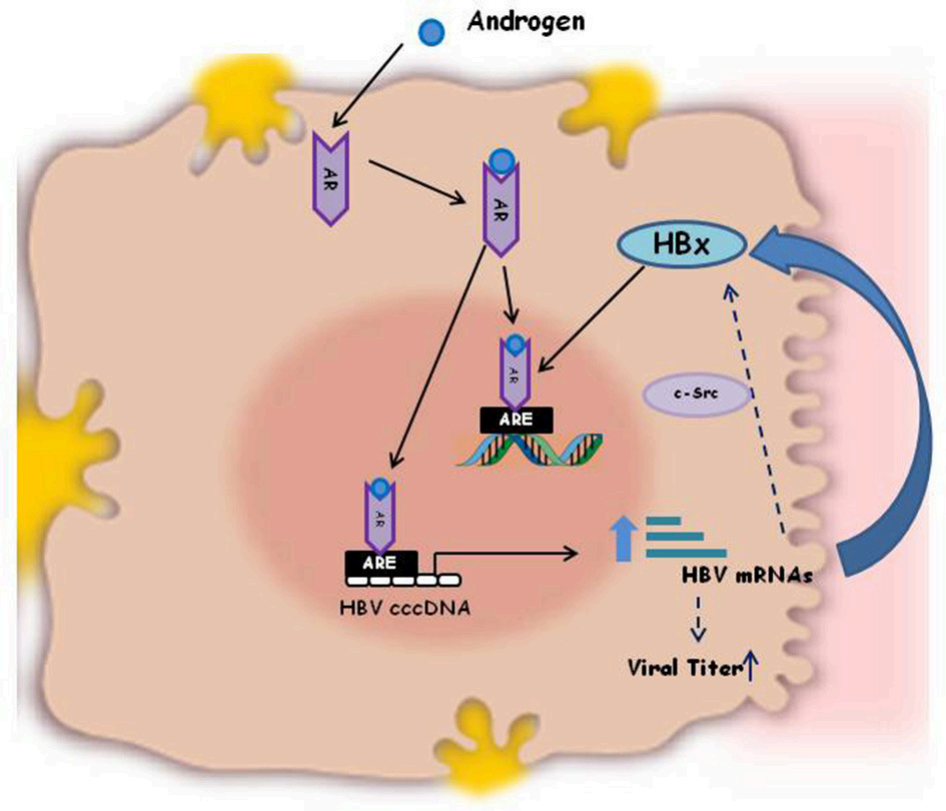
Schematic illustration of the mechanism of Androgen activation of HBV replication. HBV genome integrated in the host cell DNA contains two Androgen Responsive Elements (ARE) within the enhancer I region. The AR-Androgen complex binds either to the nuclear and viral ARE sequences, thus activating HBV genome transcription and production of the viral HBx oncoprotein. HBx, in turn, enhances AR dimerization and androgen-stimulated AR activity, through the c-Src kinase. This positive feedback loop is one of the molecular explanations for the increased HBV titers in male patients compared to females and for the different outcome of HBV infection in men and women.

Besides the effect on immune responses sex hormones can also directly influence virus activity, in some instances, being some viruses directly responsive to male or female sex hormones. As a consequence the viral load and the outcome of several viral infections are different in male and female individuals (44–50). In general HBV surface antigen (HBsAg) circulates at higher level in serum of male adult mice than in female (34) and its level decreases upon castration of the animals, thus indicating that viral antigen expression and viral replication are regulated by androgens (35). The androgens do their biological functions by binding to their cognate receptor (androgen receptor, AR), that dimerizes and translocates into the cell nucleus, where it binds to the cellular DNA, to specific Androgen Responsive Elements (ARE). This binding activates the transcriptional expression of various target genes that are associated with male phenotype. The HBV genome integrated into the host cell DNA contains two ARE elements within the enhancer I region. When the AR-Androgen complex is internalized into the hepatocytes it binds either to the nuclear and viral ARE sequences, thus activating HBV genome transcription and production of the HBV X protein (Figure 1). This latter, in turn, facilitates dimerization of the AR and enhances AR transactivation activity, through activation of Src kinase activity, thus establishing a positive feedback loop that can promote hepatocarcinogenesis. The AR further acts in conjunction with other molecules, such as cell cycle-related kinases (CCRK) that in turn activates oncogenic β-catenin in hepatocytes. This mechanism indicates that Androgen/AR signaling may promote HBV-related HCC development and explains the higher frequency of HCC as well as the higher HBV titers in serum of male sex than in female (51). Conversely, the estrogens signaling has been reported to probably suppress hepatocarcinogenesis and to be protective against HBV associated progression to HCC. The molecular mechanism for estrogen is mediated through binding of estradiol to the nuclear estrogen receptor-alfa, that inhibits the HBV enhancer I and transcription of the integrated viral genomes (51, 52).

HCV infection causes, in 80% of cases, chronic infection that usually is asymptomatic and lasts lifetime, but in a small percentage of patients can evolve to fibrosis, cirrhosis and the end stage HCC. The pathogenic mechanisms involved in the establishment of HCV chronicity and in the disease outcome are only partially described. Several factors, like age, gender, alcohol consumption, body mass index and HIV or HBV co-infection may be involved (32).

Epidemiological and clinical reports have indicated that chronic HCV infections are more prevalent in men than in women and HCV-associated disease progression to fibrosis, cirrhosis to the end- stage HCC is more rapid and more common in male patients than in female (53). Women, on the other side, are more likely to clear the virus spontaneously, after initial infection. Host factors, such as Il-28 genotype and virus genotype 1a, together with female sex have been reported to be predictor of spontaneous viral clearance of acute HCV infection (54).

A strong and efficient innate inflammatory response is considered necessary for spontaneous clearance of HCV. A critical role in host innate immune response to HCV is played by TRL7, whose activation leads to IFN-α induction and interferon-stimulated genes (ISGs) expression, subsequent to janus kinase (JAK)/signal transducer and activator of transcription (STAT) pathway induction by IFN-α (55–57). Activation of TRL7 by a synthetic ligand (SM360320) has been reported to induce HCV-specific immune response and to decrease HCV RNA levels in a replicon system *in vitro* (58); furthermore, treatment with isatoribine, a selective TLR7 agonist, caused a significant drop in plasma HCV levels in chronic HCV patients (59).

Little is known regarding the influence of sex on TRL7 expression and activation during HCV infection. A study conducted on a Maroccan chronic hepatitis C patients has reported a higher rate of spontaneous HCV clearance in women than in men, due to a particular polymorphism in TLR7 gene (60). Expression of MxA gene, one of the ISGs induced byTLR7, has been found higher in premenopausal women compared to both postmenopausal women and men (57). In addition, TLR7 activation by synthetic agonists induces a significantly higher IFN-α production in healthy women than in men (61).

However, women in postmenopausal period, when estrogens levels significantly decrease, have been reported to experience more rapid progression of hepatic fibrosis and HCC development (62) and lower response to antiviral therapy (63). Thus, pointing out for an important role of the estrogen level in determining the fate of HCV infection in female subjects and also pointing to the age effect on HCV pathogenesis.

Normal liver express estrogen receptor of both type, alfa and beta (ERα and ERβ), thus it is responsive to the estrogenic stimulus, however normal male livers have higher expression of ERα with respect to normal female livers. In contrast, in HCV associated cirrhosis and HCC ERα level has been reported to decrease only in male patients compared to normal male livers. The Authors correlated the ERα changes associated to HCV disease with the increase of inflammation markers and proliferation that are involved in the pathogenesis of liver cirrhosis and HCC, thus explaining the worse outcome of chronic HCV infection in male patients than in female (64).

The worse outcome of HCV infection in men may also be explained by the direct influence of sex hormones on HCV itself. 17β-estradiol was found to inhibit production of mature HCV virions, through ERα binding (65, 66) and to inhibit HCV entry, through down-regulation of occludin (one of the receptors used by HCV to access the hepatocytes), in infected cell cultures (67).

Studies analyzing the effect of testosterone on HCV replication are lacking so far. However, it was reported an increased expression of scavenger receptors, which are necessary for viral entry, in both HepG2 cell lines and human macrophages treated with testosterone (68, 69). Interestingly, estrogen decreased the expression of hepatic scavenger receptors in rat livers (70). The different effect of estrogens and testosterone on HCV replication may explain, at least in part, the lower incidence of HCV infection and the less common progression of liver disease to cirrhosis and HCC, in premenopausal women than in postmenopausal women and in men.

## Conclusions and future directions

From the above, the direct and diverse effects of male and female sex hormones on HBV virus genome replication and on HCV disease progression, act jointly to the effect of sex hormones on the anti-viral immune response, thus favoring the hypothesis of an interplayed action among sex-hormones, virus and immune system that determines the sex-dependent final outcome of chronic infections of hepatitis viruses. The data available to date, on the potential mechanisms determining the different susceptibility and outcome of HBV and HCV infections, either immunologic and hormonal, are fragmented and not exhaustive, but it is encouraging the disclosure in order to identify sex-specific molecular pathways involved. Molecular mechanisms of sex bias in infectious diseases is in its infancy, identification of the key players in sex-related outcome of hepatitis and of the molecular factors involved, will provide disclosure of new targets to personalized medicine and vaccinology.

## Author contributions

AR conceived and wrote the manuscript, with the support of SA. MCG contributed to write the paragraph on immune responses to hepatitis viruses.

### Conflict of interest statement

The authors declare that the research was conducted in the absence of any commercial or financial relationships that could be construed as a potential conflict of interest.

## References

[1] Soldin OP Mattison DR. Sex differences in pharmacokinetics and pharmacodynamics. Clin Pharmacokinet. (2009) 48:143–57. 10.2165/00003088-200948030-00001 19385708 PMC3644551

[2] Mazure CM Jones DP. Twenty years and still counting: including women as participants and studying sex and gender in biomedical research. BMC Womens Health (2015) 15:94. 10.1186/s12905-015-0251-9 26503700 PMC4624369

[3] Baggio G Corsini A Floreani A Giannini S Zagonel V. Gender medicine: a task for the third millennium. Clin Chem Lab Med. (2013) 51:713–27. 10.1515/cclm-2012-0849 23515103

[4] Ghosh S Klein RS. Sex drives dimorphic immune responses to viral infections. J Immunol. (2017) 198:1782–90. 10.4049/jimmunol.1601166 28223406 PMC5325721

[5] Giefing-Kröll C Berger P Lepperdinger G Grubeck-Loebenstein B. How sex and age affect immune responses, susceptibility to infections, and response to vaccination. Aging Cell (2015) 14:309–21. 10.1111/acel.12326 25720438 PMC4406660

[6] Ruggieri A Anticoli S D'Ambrosio A Giordani L Viora M. The influence of sex and gender on immunity, infection and vaccination. Ann Ist Super Sanita (2016) 52:198–204. 10.4415/ANN_16_02_11 27364394

[7] Melgert BN Oriss TB Qi Z Dixon-McCarthy B Geerlings M Hylkema MN. Macrophages: regulators of sex differences in asthma? Am J Respir Cell Mol Biol. (2010) 42:595–603. 10.1165/rcmb.2009-0016OC 19574533 PMC2874445

[8] Klein SL. Immune cells have sex and so should journal articles. Endocrinology (2012) 153:2544–50. 10.1210/en.2011-2120 22434079 PMC3359602

[9] Klein SL. Sex influences immune responses to viruses, and efficacy of prophylaxis and treatments for viral diseases. Bioessays (2012) 34:1050–9. 10.1002/bies.201200099 23012250 PMC4120666

[10] Klein SL Marriott I Fish EN. Sex-based differences in immune function and responses to vaccination. Trans R Soc Trop Med Hyg. (2015) 109:9–15. 10.1093/trstmh/tru167 25573105 PMC4447843

[11] Donnelly CA Bartley LM Ghani AC Le Fevre AM Kwong GP Cowling BJ. Gender difference in HIV-1 RNA viral loads. HIV Med. (2005) 6:170–8. 10.1111/j.1468-1293.2005.00285.x 15876283

[12] Hsieh AR Fann CS Yeh CT Lin HC Wan SY Chen YC. Effects of sex and generation on hepatitis B viral load in families with hepatocellular carcinoma. World J Gastroenterol. (2017) 23:876–84. 10.3748/wjg.v23.i5.876 28223732 PMC5296204

[13] Park HJ Choi JM. Sex-specific regulation of immune responses by PPARs. Exp Mol Med. (2017) 49:e364. 10.1038/emm.2017.102 28775365 PMC5579504

[14] Hewagama A Patel D Yarlagadda S Strickland FM Richardson BC. Stronger inflammatory/cytotoxic T-cell response in women identified by microarray analysis. Genes Immun. (2009) 10:509–16. 10.1038/gene.2009.12 19279650 PMC2735332

[15] Patin E Hasan M Bergstedt J Rouilly V Libri V Urrutia A Natural variation in the parameters of innate immune cells is preferentially driven by genetic factors. Nat Immunol. (2018) 19:302–14. 10.1038/s41590-018-0049-7 29476184

[16] Aguirre-Gamboa R Joosten I Urbano PCM van der Molen RG van Rijssen E van Cranenbroek B. Differential effects of environmental and genetic factors on T and B Cell immune traits. Cell Rep. (2016) 17:2474–87. 10.1016/j.celrep.2016.10.053 27818087 PMC5130901

[17] Bhatia A Sekhon HK Kaur G. Sex hormones and immune dimorphism. Sci World J. (2014) 2014:159150. 10.1155/2014/159150 25478584 PMC4251360

[18] Kovats S Carreras E Agrawal H Sex steroid receptors in immune cells. In: Klein SL Roberts CW, editors. Sex Hormones And Immunity to Infection. Berlin: Springer-Verlag (2010). p. 53–92.

[19] Khan D Ansar Ahmed S. The immune system is a natural target for estrogen action: opposing effects of estrogen in two prototypical autoimmune diseases. Front Immunol. (2016) 6:635. 10.3389/fimmu.2015.00635 26779182 PMC4701921

[20] Foo YZ Nakagawa S Rhodes G Simmons LW. The effects of sex hormones on immune function: a meta-analysis. Biol Rev Camb Philos Soc. (2017) 92:551–71. 10.1111/brv.12243 26800512

[21] Trigunaite A Dimo J Jørgensen TN. Suppressive effects of androgens on the immune system. Cell Immunol. (2015) 294:87–94. 10.1016/j.cellimm.2015.02.004 25708485

[22] D'Agostino P Milano S Barbera C Di Bella G La Rosa M Ferlazzo V. Sex hormones modulate inflammatory mediators produced by macrophages. Ann NY Acad Sci. (1999) 876:426–9. 10.1111/j.1749-6632.1999.tb07667.x 10415638

[23] Liva SM Voskuhl RR. Testosterone acts directly on CD4^+^ T lymphocytes to increase IL-10 production. J Immunol. (2001) 167:2060–7. 10.4049/jimmunol.167.4.2060 11489988

[24] Pergola C Dodt G Rossi A Neunhoeffer E Lawrenz B Northoff H. ERK-mediated regulation of leukotriene biosynthesis by androgens: a molecular basis for gender differences in inflammation and asthma. Proc Natl Acad Sci USA. (2008) 105:19881–6. 10.1073/pnas.0809120105 19064924 PMC2597692

[25] Malkin CJ Pugh PJ Jones RD Kapoor D Channer KS Jones TH. The effect of testosterone replacement on endogenous inflammatory cytokines and lipid profiles in hypogonadal men. J Clin Endocrinol Metab. (2004) 89:3313–8. 10.1210/jc.2003-031069 15240608

[26] Bobjer J Katrinaki M Tsatsanis C Lundberg Giwercman Y Giwercman A. Negative association between testosterone concentration and inflammatory markers in young men: a nested cross-sectional study. PLoS ONE (2013) 8:e61466. 10.1371/journal.pone.0061466 23637840 PMC3630214

[27] Kissick HT Sanda MG Dunn LK Pellegrini KL On ST Noel JK. Androgens alter T-cell immunity by inhibiting T-helper 1 differentiation. Proc Natl Acad Sci USA. (2014) 111:9887–92. 10.1073/pnas.1402468111 24958858 PMC4103356

[28] Kumar N Shan LX Hardy MP Bardin CW Sundaram K. Mechanism of androgen-induced thymolysis in rats. Endocrinology (1995) 136:4887–93. 10.1210/endo.136.11.7588221 7588221

[29] Seillet C Laffont S Trémollières F Rouquié N Ribot C Arnal JF. The TLR-mediated response of plasmacytoid dendritic cells is positively regulated by estradiol *in vivo* through cell-intrinsic estrogen receptor α signaling. Blood (2012) 119:454–64. 10.1182/blood-2011-08-371831 22096248

[30] Tai P Wang J Jin H Song X Yan J Kang Y. Induction of regulatory T cells by physiological level estrogen. J Cell Physiol. (2008) 214:456–64. 10.1002/jcp.21221 17654501

[31] Lü FX Abel K Ma Z Rourke T Lu D Torten J. The strength of B cell immunity in female rhesus macaques is controlled by CD8^+^ T cells under the influence of ovarian steroid hormones. Clin Exp Immunol. (2002) 128:10–20. 10.1046/j.1365-2249.2002.01780.x 11982585 PMC1906365

[32] Shlomai A de Jong YP Rice CM. Virus associated malignancies: the role of viral hepatitis in hepatocellular carcinoma. Semin Cancer Biol. (2014) 26:78–88. 10.1016/j.semcancer.2014.01.004 24457013 PMC4048791

[33] Ruggieri A Barbati C Malorni W. Cellular and molecular mechanisms involved in hepatocellular carcinoma gender disparity. Int J Cancer (2010) 127:499–504. 10.1002/ijc.25298 20201099

[34] Farza H Salmon AM Hadchouel M Moreau JL Babinet C Tiollais P. Hepatitis B surface antigen gene expression is regulated by sex steroids and glucocorticoids in transgenic mice. Proc Natl Acad Sci USA. (1987) 84:1187–91. 10.1073/pnas.84.5.1187 3469661 PMC304391

[35] Breidbart S Burk RD Saenger P. Hormonal regulation of hepatitis B virus gene expression: influence of androgen receptor. Pediatr Res. (1993) 34:300–2. 10.1203/00006450-199309000-00012 8134171

[36] Tsay PK Tai DI Chen YM Yu CP Wan SY Shen YJ. Impact of gender, viral transmission and aging in the prevalence of hepatitis B surface antigen. Chang Gung Med J. (2009) 32:155–64. 19403005

[37] Yu MW Chen CJ. Elevated serum testosterone levels and risk of hepatocellular carcinoma. Cancer Res. (1993) 53:790–4. 8381328

[38] Su FH Chen JD Cheng SH Lin CH Liu YH Chu FY. Seroprevalence of Hepatitis-B infection amongst Taiwanese university students 18 years following the commencement of a national Hepatitis-B vaccination program. J Med Virol. (2007) 79:138–43. 10.1002/jmv.20771 17177303

[39] Alward WL McMahon BJ Hall DB Heyward WL Francis DP Bender TR. The long-term serological course of asymptomatic hepatitis B virus carriers and the development of primary hepatocellular carcinoma. J Infect Dis. (1985) 151:604–9. 10.1093/infdis/151.4.604 2982971

[40] Kosinska AD Pishraft-Sabet L Wu W Fang Z Lenart M Chen J. Low hepatitis B virus-specific T-cell response in males correlates with high regulatory T-cell numbers in murine models. Hepatology (2017) 66:69–83. 10.1002/hep.29155 28295453

[41] Yan Z Tan W Dan Y Zhao W Deng C Wang Y. Estrogen receptor alpha gene polymorphisms and risk of HBV-related acute liver failure in the Chinese population. BMC Med Genet. (2012) 13:49. 10.1186/1471-2350-13-49 22727021 PMC3412699

[42] Naugler WE Sakurai T Kim S Maeda S Kim K Elsharkawy AM. Gender disparity in liver cancer due to sex differences in MyD88-dependent IL-6 production. Science (2007) 317:121–4. 10.1126/science.1140485 17615358

[43] Prieto J. Inflammation, HCC and sex: IL-6 in the centre of the triangle. J Hepatol. (2008). 48:380–1. 10.1016/j.jhep.2007.11.007 18093689

[44] Scully EP. Sex differences in HIV infection. Curr HIV/AIDS Rep. (2018) 15:136–46. 10.1007/s11904-018-0383-2 29504062 PMC5882769

[45] Ziegler SM Altfeld M. Human immunodeficiency virus 1 and Type I interferons-where sex makes a difference. Front Immunol. (2017) 8:1224. 10.3389/fimmu.2017.01224 29033943 PMC5625005

[46] Hassan MM Botrus G Abdel-Wahab R Wolff RA Li D Tweardy D. Estrogen replacement reduces risk and increases survival times of women with hepatocellular carcinoma. Clin Gastroenterol Hepatol. (2017) 15:1791–9. 10.1016/j.cgh.2017.05.036 28579181 PMC5901750

[47] Weyn C Vanderwinden JM Rasschaert J Englert Y Fontaine V. Regulation of human papillomavirus type 16 early gene expression in trophoblastic and cervical cells. Virology (2011) 412:146–55. 10.1016/j.virol.2010.12.056 21276600

[48] Itaborahy RM Mancini DA de Medeiros SF. Response to the influenza vaccine based on estradiol use in menopausal women. Vaccine (2016) 34:1358–62. 10.1016/j.vaccine.2016.01.052 26851841

[49] Peretz J Pekosz A Lane AP Klein SL Estrogenic compounds reduce influenza A virus replication in primary human nasal epithelial cells derived from female, but not male, donors. Am J Physiol Lung Cell Mol Physiol. (2016) 310:L415–25. 10.1152/ajplung.00398.2015 26684252 PMC4773846

[50] Furman D Hejblum BP Simon N Jojic V Dekker CL Thiébaut R. Systems analysis of sex differences reveals an immunosuppressive role for testosterone in the response to influenza vaccination. Proc Natl Acad Sci USA. (2014) 111:869–74. 10.1073/pnas.1321060111 24367114 PMC3896147

[51] Wang SH Yeh SH Lin WH Wang HY Chen DS Chen PJ. Identification of androgen response elements in the enhancer I of hepatitis B virus: a mechanism for sex disparity in chronic hepatitis B. Hepatology (2009) 50:1392–402. 10.1002/hep.23163 19670412

[52] Wang SH Chen PJ Yeh SH. Gender disparity in chronic hepatitis B: mechanisms of sex hormones. J Gastroenterol Hepatol. (2015) 30:1237–45. 10.1111/jgh.12934 25708186

[53] Di Martino V Lebray P Myers RP Pannier E Paradis V Charlotte F. Progression of liver fibrosis in women infected with hepatitis C: long-term benefit of estrogen exposure. Hepatology (2004) 40:1426–33. 10.1002/hep.20463 15565616

[54] Grebely J Page K Sacks-Davis R van der Loeff MS Rice TM Bruneau J. The effects of female sex, viral genotype, and IL28B genotype on spontaneous clearance of acute hepatitis C virus infection. Hepatology (2014) 59:109–20. 10.1002/hep.26639 23908124 PMC3972017

[55] Chang S Kodys K Szabo G. Impaired expression and function of toll-like receptor 7 in hepatitis C virus infection in human hepatoma cells. Hepatology (2010) 51:35–42. 10.1002/hep.23256 19821521

[56] Zhang YL Guo YJ Bin Li Sun SH. Hepatitis C virus single- stranded RNA induces innate immunity via toll-like receptor 7. J Hepatol. (2009) 51:29–38. 10.1016/j.jhep.2009.03.012 19443072

[57] Mekky RY Hamdi N El-Akel W Esmat G Abdelaziz AI. Estrogen-related MxA transcriptional variation in hepatitis C virus-infected patients. Transl Res. (2012) 159:190–6. 10.1016/j.trsl.2011.08.002 22340769

[58] Lee J Wu CC Lee KJ Chuang TH Katakura K Liu YT. Activation of anti-hepatitis C virus responses via toll-like receptor 7. Proc Natl Acad Sci USA. (2006) 103:1828–33. 10.1073/pnas.0510801103 16446426 PMC1413670

[59] Horsmans Y Berg T Desager JP Mueller T Schott E Fletcher SP. Isatoribine, an agonist of TLR7, reduces plasma virus concentration in chronic hepatitis C infection. Hepatology (2005) 42:724–31. 10.1002/hep.20839 16116638

[60] Fakhir FZ Lkhider M Badre W Alaoui R Meurs EF Pineau P. Genetic variations in toll-like receptors 7 and 8 modulate natural hepatitis C outcomes and liver disease progression. Liver Int. (2018) 38:432–42. 10.1111/liv.13533 28752959

[61] Berghöfer B Frommer T Haley G Fink L Bein G Hackstein H. TLR7 ligands induce higher IFN-alpha production in females. J Immunol. (2006) 177:2088–96. 16887967 10.4049/jimmunol.177.4.2088

[62] Villa E Karampatou A Cammà C Di Leo A Luongo M Ferrari A. Early menopause is associated with lack of response to antiviral therapy in women with chronic hepatitis C. Gastroenterology (2011) 140:818–29. 10.1053/j.gastro.2010.12.027 21167831

[63] Yu JW Sun LJ Zhao YH Kang P Yan BZ. Impact of sex on virologic response rates in genotype 1 chronic hepatitis C patients with peginterferon alpha-2a and ribavirin treatment. Int J Infect Dis. (2011) 15:e740–6. 10.1016/j.ijid.2011.05.018 21803628

[64] Iyer JK Kalra M Kaul A Payton ME Kaul R. Estrogen receptor expression in chronic hepatitis C and hepatocellular carcinoma pathogenesis. World J Gastroenterol. (2017) 23:6802–16. 10.3748/wjg.v23.i37.6802 29085224 PMC5645614

[65] Hayashida K Shoji I Deng L Jiang DP Ide YH Hotta H. 17β-estradiol inhibits the production of infectious particles of hepatitis C virus. Microbiol Immunol. (2010) 54:684–90. 10.1111/j.1348-0421.2010.00268.x 21044142

[66] Magri A Barbaglia MN Foglia CZ Boccato E Burlone ME Cole S. 17, β-estradiol inhibits hepatitis C virus mainly by interference with the release phase of its life cycle. Liver Int. (2017) 37:669–77. 10.1111/liv.13303 27885811 PMC5448036

[67] Ulitzky L Lafer MM KuKuruga MA Silberstein E Cehan N Taylor DR. A new signaling pathway for HCV inhibition by estrogen: GPR30 activation leads to cleavage of occludin by MMP-9. PLoS ONE (2016) 11:e0145212. 10.1371/journal.pone.0145212 26731262 PMC4701175

[68] Scarselli E Ansuini H Cerino R Roccasecca RM Acali S Filocamo G. The human scavenger receptor class B type I is a novel candidate receptor for the hepatitis C virus. EMBO J. (2002):5017–25. 10.1093/emboj/cdf529 12356718 PMC129051

[69] Langer C Gansz B Goepfert C Engel T Uehara Y von Dehn G. Testosterone up-regulates scavenger receptor BI and stimulates cholesterol efflux from macrophages. Biochem Biophys Res Commun. (2002) 296:1051–7. 10.1016/S0006-291X(02)02038-7 12207878

[70] Stangl H Graf GA Yu L Cao G Wyne K. Effect of estrogen on scavenger receptor BI expression in the rat. J Endocrinol. (2002) 175:663–72. 10.1677/joe.0.1750663 12475377

